# Crotoxin-Induced Mice Lung Impairment: Role of Nicotinic Acetylcholine Receptors and COX-Derived Prostanoids

**DOI:** 10.3390/biom10050794

**Published:** 2020-05-20

**Authors:** Marco Aurelio Sartim, Camila O. S. Souza, Cassiano Ricardo A. F. Diniz, Vanessa M. B. da Fonseca, Lucas O. Sousa, Ana Paula F. Peti, Tassia Rafaella Costa, Alan G. Lourenço, Marcos C. Borges, Carlos A. Sorgi, Lucia Helena Faccioli, Suely Vilela Sampaio

**Affiliations:** 1 Department of Clinical Analysis, Toxicology and Food Sciences, School of Pharmaceutical Sciences of Ribeirão Preto, University of São Paulo, Ribeirão Preto 14040-903, SP, Brazil; marcosartim@hotmail.com (M.A.S.); camila.oliveirasilva@usp.br (C.O.S.S.); losousa@usp.br (L.O.S.); anaferranti@usp.br (A.P.F.P.); tassiarcosta@yahoo.com.br (T.R.C.); sorgi@fcfrp.usp.br (C.A.S.); faccioli@fcfrp.usp.br (L.H.F.); 2 Department of Pharmacology, Ribeirão Preto Medical School, University of São Paulo, Ribeirão Preto 14049-900, SP, Brazil; crafd87@gmail.com; 3 Department of Internal Medicine, Ribeirão Preto Medical School, University of São Paulo, Ribeirão Preto 14049-900, SP, Brazil; van.macielfonseca@gmail.com (V.M.B.d.F.); marcosborges@fmrp.usp.br (M.C.B.); 4 Department of Basic and Oral Biology, School of Dentistry of Ribeirão Preto, University of São Paulo, Ribeirão Preto 14040-904, SP, Brazil; lourenco@forp.usp.br

**Keywords:** crotoxin, snake venom, lung impairment, inflammatory response, lipid mediators, neuromuscular blocker

## Abstract

Respiratory compromise in *Crotalus durissus terrificus* (C.d.t.) snakebite is an important pathological condition. Considering that crotoxin (CTX), a phospholipase A_2_ from C.d.t. venom, is the main component of the venom, the present work investigated the toxin effects on respiratory failure. Lung mechanics, morphology and soluble markers were evaluated from Swiss male mice, and mechanism determined using drugs/inhibitors of eicosanoids biosynthesis pathway and autonomic nervous system. Acute respiratory failure was observed, with an early phase (within 2 h) characterized by enhanced presence of eicosanoids, including prostaglandin E2, that accounted for the increased vascular permeability in the lung. The alterations of early phase were inhibited by indomethacin. The late phase (peaked 12 h) was marked by neutrophil infiltration, presence of pro-inflammatory cytokines/chemokines, and morphological alterations characterized by alveolar septal thickening and bronchoconstriction. In addition, lung mechanical function was impaired, with decreased lung compliance and inspiratory capacity. Hexamethonium, a nicotinic acetylcholine receptor antagonist, hampered late phase damages indicating that CTX-induced lung impairment could be associated with cholinergic transmission. The findings reported herein highlight the impact of CTX on respiratory compromise, and introduce the use of nicotinic blockers and prostanoids biosynthesis inhibitors as possible symptomatic therapy to *Crotalus durissus terrificus* snakebite.

## 1. Introduction

Rattlesnakes are native to the Americas and are responsible for several cases of envenomation in the continent [1]. They caused 56.3% of the ophidic accidents in North America, while snakes from genus *Crotalus*—the major rattlesnake genus in Central and South Americas—caused less than 10% of the 50,000 cases of snakebite per year in the region [1,2,3,4]. In Brazil, the 2016 report from the Ministry of Health [5] recorded that snakes from genus *Crotalus* participated in approximately 10% of the notified accidents caused by venomous snakes, and accounted for the highest mortality rate.

Typical clinical manifestations during envenomation are related to severe systemic disturbances, such as neurotoxicity, coagulation alterations, and respiratory and renal failure associated with myotoxicity, leading to failure of end-organs and death [6,7]. Although rarely reported, respiratory impairment induced by rattlesnake bite is a potential lethal complication associated with severe cases of envenomation [8,9,10,11,12], and it is characterized by airway obstruction, bronchospasm, soft tissue edema, or subjective symptoms including throat tightening and nasal congestion [11]. *Crotalus durissus* snakebite causes other respiratory abnormalities within the first 48 h, such as dyspnea, tachypnea, use of accessory muscles of respiration and flaring of the nostrils, followed by decreased blood pH and pO_2_, and increased pCO_2_ levels [9]. *Crotalus durissus terrificus* (C.d.t.) [13] and *Crotalus durissus cascavella* [14] crude venom induces similar respiratory disturbances in a mice model of envenomation, in addition to (i) mechanical alterations in lung tissues characterized by increased lung static- and dynamic-elastance, and resistive- and viscoelastic-pressure; and (ii) morphological alterations including increased leukocyte infiltration, hemorrhage, and edema [13,14].

Crotoxin (CTX) is the main toxic component of the venom from the South American rattlesnake C.d.t. This toxin is isolated as a heterodimeric complex composed of a basic enzymatically active phospholipase A_2_ (CB) non-covalently bound to an acidic non-enzymatic domain (crotapotin) [15,16,17]. CTX has been associated with several pathological conditions such as neurotoxicity, myotoxicity, and immune alterations [18,19,20,21,22], but its participation in respiratory disturbances is poorly reported and remains controversial. The CTX complex (CB/crotapotin), but not its components alone (CB or crotapotin), causes complete respiratory arrest associated with decreased blood pH and pO_2_, and increased pCO_2_ in rabbits [23]. In contrast, CTX do not modulate respiration frequency and amplitude in dogs [24].

The reported clinical and experimental data on C.d.t. effects on respiratory function stress the importance of investigating how CTX, the most abundant venom toxin, participates in the impairment of lung physiology. Literature reports are limited, do not show lung pathological alterations in depth, and do not elucidate the mechanism by which CTX acts. In this sense, the present work investigated the pathophysiology of CTX-induced lung disturbances in mice, in particular the morphological and functional alterations, as well as the participation of peripheral nervous system and production of lipid mediators during respiratory failure.

## 2. Materials and Methods

### 2.1. Animals

Male 8–9 week-old Swiss mice (35–40 g) were provided by the Central Animal Facility of the University of São Paulo, Campus of Ribeirão Preto (Ribeirão Preto, SP, Brazil). The animals were housed at Animal Facility at Pharmaceutical Sciences School of Ribeirão Preto (FCFRP–USP) under controlled conditions of temperature (23 °C) and brightness (12 h light/dark cycles), and with free access to food and water. The experiments were performed at FCFRP-USP following animal care procedures, which experimental protocols are in accordance with the COBEA (Brazilian College of Animal Experimentation) guidelines and were approved by the Ethics Committee on Animal Use (CEUA) from the University of São Paulo, Campus of Ribeirão Preto (protocol number: 15.1.807.60.1).

### 2.2. Crotoxin

Crotoxin (CTX) was isolated from C.d.t crude venom as described by Muller and colleagues [1]. To eliminate endotoxin contaminants, CTX sample was purified using Affi-Prep Polymyxin Resin according to the manufacturer’s instructions (Bio-Rad—Hercules, CA, USA). The endotoxin levels were lower than 0.01 EU/µg of CTX (1 EU = 0.1 ng of endotoxin), as determined using the limulus amoebocyte lysate kit (Lonza Biosciences—Walkersville, MD, USA). Protein concentration in CTX samples was quantified using the BCA kit, according to the manufacturer’s instructions (Thermo Scientific—Rockford, IL, USA).

### 2.3. CTX and Drug Treatments

#### 2.3.1. CTX Dose- and Time-Response Experiments

To select a suitable CTX dose for the in vivo assays, a dose-response experiment was carried out using subcutaneous injection (s.c.) of CTX at 10–300 µg/Kg or saline (control). After 6 h, mice were anesthetized with intraperitoneal (i.p.) injection of ketamine/xylazine solution (80/10 mg/kg), their lung and heart were removed for analysis, and their blood was collected by cardiac puncture for analysis of whole blood and serum. Based on the survival profile of the animals, the CTX dose of 300 µg/Kg s.c. and time treatments of 2, 6, and 12 h were selected for further experiments. Animals treated with saline under the same conditions were used as the control group.

#### 2.3.2. Drug Treatments

Drugs that act as antagonists or inhibitors of eicosanoids production and peripheral neuronal pathways were used to investigate the toxicological mechanisms of CTX action in mice. Indomethacin (Sigma-Aldrich—St. Louis, MO, USA) was administered (3 mg/Kg i.p.) 4.5 h before CTX s.c. injection [2,3]. MK-591 (AdooQ Bioscience—Irvine, CA, USA) was administered (40 mg/Kg i.p.) 30 min before CTX s.c. injection [4]. Hexamethonium bromide (Sigma-Aldrich) was administered intravenously (i.v. tail vein) at a dose of 10 mg/Kg, 15 min before CTX s.c. injection [5]. Methyl-atropine (Sigma-Aldrich) was administered (30 mg/Kg i.p.) 30 min before CTX s.c. injection [6]. Neostigmine (Sigma-Aldrich) was administered (0.1 mg/Kg i.p.) 10 min before CTX s.c. injection [7]. Propranolol (Tocris Bioscience—Bristol, UK) was administered (5 mg/Kg i.p.) 30 min before CTX s.c. injection [8]. Indomethacin was prepared in Tris-HCl 100 mM pH 8.2, while the other drugs were prepared in saline (0.9% NaCl).

### 2.4. In Vivo Experiments

**Scheme 1:**
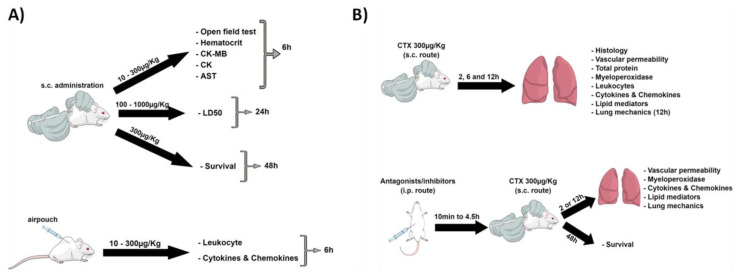
Experimental protocol ratilonale. (**A**) Determination of crotoxin (CTX) working dose. (**B**) CTX-induced lung alterations.

In order to better illustrate the experimental protocol rationale, a scheme was performed (Scheme 1).

#### 2.4.1. Lethality

To determine the median lethal dose (LD_50_), CTX was administered to mice at doses ranging from 100 to 1000 µg/Kg (s.c.), and the animal survival rate was analyzed after 24 h. To analyze the survival rate profile, the toxin was administered at a dose of 300 µg/Kg (s.c.) and the survival rate was monitored for 144 h (6 days). To examine the effect of pharmacological antagonists on the survival rate of CTX-treated animals, the drugs were administered before CTX (300 µg/Kg s.c.) as described in the previous section, and the survival rate was monitored every 12 h during 48 h (2 days).

#### 2.4.2. Open Field Test

The open field test was performed in an independent experimental group to evaluate the CTX-induced locomotor effects, as previously described [9]. Mice were placed individually at the center of a circular open-field arena (40 cm diameter) divided into quadrants where the exploratory activity was videotaped during 6 min. A trained experimenter manually counted the number of quadrants crossed in the last 4 min.

#### 2.4.3. Vascular Permeability

The Evans blue permeability assay was performed as described elsewhere [10]. Thirty minutes before the end of the experimental period, 200 µL of 0.5% Evans blue solution in sterile saline were injected into the mice tail vein. Next, the animals were euthanized and their left lung lobe and heart were collected, weighted, and immersed in 500 µL of formamide for 24 h, at 55 °C, to extract Evans blue. Finally, absorbance of the supernatant was recorded at 610 nm and the amount of extravasated dye per mg of organ was calculated from the Evans blue standard curve.

#### 2.4.4. Air Pouch Model to Examine Local Inflammation

To examine the CTX-induced local inflammation, we used the dorsal air pouch model. First, mice were anesthetized with ketamine/xylazine solution (80/10 mg/kg, i.p.), and the midline of their dorsal region was shaved. Approximately 3 mL of sterile air were injected subcutaneously with a 25 G needle, through a sterile 0.22 µm filter (Millex, Merck Millipore—Burlington, VT, USA). At the third day, a second boost of 2 mL of sterile air was injected into the pre-existing air pouch. At the sixth day, 0.5 mL of CTX (10–300 µg/Kg) or saline (control) were injected into the air pouch, followed by injection of 2 mL of incomplete RPMI medium. The pouch fluid was collected and stained with Trypan Blue for total leukocyte counting, using the Countess II automated cell counter (Life Technologies—Carlsbad, CA, USA). Differential leukocyte counts were performed on cytospin preparations of pouch fluid stained with Panoptic kit (Laborclin—Pinhais, Brazil). Supernatant was stored at −80 °C for further quantification of cytokines and total proteins.

#### 2.4.5. Lung Mechanics

A tracheal cannula connected to a small animal FlexiVent^®^ ventilator (Scireq—Montreal, QC, Canada) was inserted into mice anesthetized with ketamine/xylazine (100/10 mg/kg, i.p.), and further ventilated with respiratory frequency of 150 breaths/minute and positive end-expiratory pressure of 3 cmH_2_O. Pancuronium bromide (1.2 mg/kg i.p.) was administered for total paralysis before analysis of lung mechanical functions using the forced oscillation technique, in particular the single compartment and the constant phase model. In addition, a respiratory pressure–volume curve was built and the quasi-static and dynamic respiratory compliance were calculated by fitting the Salazar–Knowles equation to pressure-volume curves. Results were expressed as respiratory system elastance, tissue elastance, quasi-static and dynamic respiratory compliance, respiratory system compliance, inspiratory capacity, and tissue resistance.

### 2.5. Biological Parameters and Markers

#### 2.5.1. Biochemical Markers

Mice blood samples collected without anticoagulant were kept at room temperature for 30 min to allow clotting, and centrifuged at 1300× *g* for 15 min. The resulting serum supernatant was collected and stored at −80 °C. The damage-associated serum biomarkers aspartate aminotransferase (AST), creatine kinase (CK), and creatine kinase MB (CK-MB) were quantified according to the manufacturer’s instructions (Wiener Lab—Rosário, Argentina).

#### 2.5.2. Pro-Inflammatory Cytokines

The pro-inflammatory mediators chemokine (CXC motif) ligand 1 (CXCL-1), interleukin-1β (IL-1 β), interleukin-6 (IL-6), and tumor necrosis factor-α (TNF-α) were quantified in dorsal air pouch fluid, lung homogenates and bronchoalveolar fluid (BALF) using enzyme-linked immunosorbent assay (ELISA) kits, as recommended by the manufacturer (R&D Systems—Minneapolis, MN, USA).

#### 2.5.3. Hematocrit

Hematocrit was determined in EDTA-anticoagulated whole blood samples, using the automated hematology analyzer Cell Dyn 3700 (Abbott—Chicago, IL, USA).

#### 2.5.4. Gene Expression

Expression of genes of inflammatory cytokines and enzymes involved in eicosanoid metabolism was analyzed in lung after 2 h of treatment with CTX or saline. The right lower lung lobule was harvested, weighed, and homogenized. Total RNA was extracted using PureLink RNA Mini Kit according to the manufacturer’s specifications (Invitrogen—Carlsbad, CA, USA), quantified using NanoDrop 2000 (Thermo Scientific)—considering the absorbance ratios A260/280 and A260/230 between 1.8–2.2—and treated with DNase I amplification grade (Invitrogen). Next, cDNA was synthesized from 2 μg of the total RNA extracted, using the High Capacity cDNA Reserve Transcription Kit (Applied Biosystems—Foster City, CA, USA).

Aliquots (40 ng) of the total cDNA were amplified by quantitative reverse transcriptase-polymerase chain reaction (qRT–PCR). Custom plates for RT^2^ PCR analysis were acquired from Applied Biosystems and contained genes of arachidonate 5-lipoxygenase-activating protein (*Alox5ap*–Mm00802100_m1), cyclooxygenase-2 (*Ptgs2*–Mm00478374_m1), cytosolic phospholipase A_2_ (*Pla2g4a*–Mm00447040_m1), interleukin-1β (*IL1b*–Mm01336189_m1), interleukin-6 (*IL6*–Mm00446191_m1), leukotriene A_4_ hydrolase (*Lta4h*–Mm01246216_m1), 5-lipoxygenase (*Alox5*–Mm01182743_m1), 12-lipoxygenase (*Alox12*–Mm00545833_m1), 15-lipoxygenase (*Alox15*–Mm01250458_m1), and tumor necrosis factor (*Tnf*–Mm00443258_m1). Glucuronidase beta (*Gusb*–Mm00446953_m1), glyceraldehyde-3-phosphate dehydrogenase (*Gapdh*–Mm99999915_g1), and hypoxanthine phosphoribosyltransferase 1 (*Hprt1*–Mm00446968_m1) were used as reference genes. PCR reactions were performed using the TaqMan Fast Universal PCR Mastermix 2X (Applied Biosystems—Austin, TX, USA) in a StepOnePlus Real-Time PCR System (Applied Biosystems), according to the manufacturer’s instructions.

Results were analyzed using the DataAssist™ v3.01 software (Applied Biosystems), and the threshold cycle (Ct) cut-off value was set up as 40. Normalization was done by subtracting the Ct mean value of the gene of interest from the Ct mean value of the three reference genes (*GAPDH, GUSB* and *HPRT1*). The values obtained for the negative control were used as reference for comparison. The relative expression of each gene was calculated by the 2^ΔΔCt^ method [11].

#### 2.5.5. Eicosanoids

The eicosanoids 11-hydroxyeicosatetraenoic acid (11-HETE), 12-hydroxyeicosatetraenoic acid (12-HETE), 15-hydroxyeicosatetraenoic acid (15-HETE), and 5-oxo-eicosatetraenoic acid (5-oxo-ETE), leukotriene B_4_ (LTB_4_), 6-*trans*-leukotriene B_4_ (6-*trans*-LTB_4_), prostaglandin B_2_ (PGB_2_), prostaglandin D_2_ (PGD_2_), prostaglandin E_2_ (PGE_2_), 6-keto-prostaglandin E_2_ (6-keto-PGE_2_), prostaglandin F_2_α (PGF_2_α), 6-keto-prostaglandin F_1_α (6-keto-PGF_1_α, the stable prostacyclin (PGI_2_) metabolite), and tromboxane B_2_ (TXB_2_) were quantified in lungs from mice treated with CTX or saline.

Left lung lobules were collected, weighed, and homogenized in incomplete RPMI 1640 medium (1 mL/100 mg lung). The lung homogenate supernatant was mixed with methanol (1:1 *v/v* final) and submitted to solid phase extraction in a C18 column for lipids extraction. A 10 µL aliquot of each sample extracted was analyzed using the liquid chromatography-tandem mass spectrometry (LC-MS/MS) system TripleTOF^®^ 5600+ (AB Sciex—Foster, CA, USA), as previously described [12]. Data were acquired using the Analyst software (SCIEX—Framingham, MA, USA), reviewed using the PeakView^TM^ software (SCIEX), and quantified using the MultiQuant^TM^ software (SCIEX).

#### 2.5.6. Myeloperoxidase Activity

Myeloperoxidase (MPO) activity was determined as described elsewhere [13]. Animals heart and mid right lung lobule were removed, weighed, and homogenized in a tissue homogenizer. MPO activity in the supernatant was determined using 3,3′,5,5′-tetramethylbenzidine (TMB) as substrate (BD Bioscience—San Jose, USA), and recording absorbance at 450 nm. The results were reported as Units (1 Unit = ∆0.1 Abs 450 nm) per mg of tissue.

### 2.6. Lung Histology

The left lung lobule was collected and fixed in a 10% formaldehyde solution in PBS pH 7.4 for 24 h. Afterwards, the samples were immersed in alcohol and xylol solutions, and included in paraffin. Sections of 4 µm were prepared using the RM-2125 microtome (Leica—Wetzlar, Germany) and stained with hematoxylin and eosin (HE). Morphological analysis was performed using the DM LB2light microscope (Leica) coupled to the DC 300F camera (Leica). The captured images were analyzed using the Leica QWin software (Leica). Quantitative histopathologic analysis of lung injury was performed using a score system based on the following criteria: leukocyte infiltration, vascular congestion, alveolar hemorrhage, and edema. Each criterion was graded on a scale from 0 to 3 (0, absent; 1, mild; 2, moderate; and 3, severe). Lung injury score was calculated for each specimen and treatment period. The pathologist who performed histological analysis was blinded to the intervention. The alveolar sac area represented by the empty space (white area) in HE-stained lung histological images was calculated using the IM-50 software (Leica).

### 2.7. Analysis of Lung Leukocyte Population

Infiltrating leukocytes were isolated from mice lung using the protocol reported by Souza and colleagues [14]. Briefly, the upper right lung lobule was collected, minced with sterile scissors in RPMI 1640 medium, and treated with digestion buffer containing 0.05 mg/mL liberase (Roche—Basel, Switzerland) and 0.5 mg/mL DNase (Sigma-Aldrich) for 45 min, at 37 °C, under shaking at 2000 rpm. Tissue debris were removed using a 100 µm cell strainer. Next, red blood cells were lysed and the remaining cells were washed with PBS, centrifuged, and suspended in RPMI 1640 containing 10% FBS. Cells were fixed using cytospin slides and stained with Panoptic kit (Laborclin—Pinhais, Brazil) to perform differential leukocyte counting. Next, neutrophil phenotypes were analyzed by flow cytometry using the Ly6G PE-Cy7 conjugated (Cat#560601, RRID:AB_1727562) and CD62L BB515 conjugated (Clone MEL-14) antibodies (BD Bioscience—San Jose, CA, USA). Data from 20,000 events were acquired using a FACSCanto II flow cytometer equipped with the FACSDiva software (BD Biosciences), and further plotted and analyzed using the FlowJo software v.10.0.7 (Tree Star, Inc.—Ashland, OR, USA).

### 2.8. Statistical Analysis

The GraphPad Prism software version 5.01 (GraphPad Software Inc.—San Diego, CA, USA) was used to plot graphics and perform statistical data analysis. The unpaired Student’s *t*-test was used to analyze differences between two groups, while one-way analysis of variance (ANOVA) followed by the Bonferroni’s post-test was used for comparison of multiple groups. The survival rate was expressed as percentage of live animals, and the Mantel–Cox log-rank test was used to compare the survival curves. Two-way ANOVA followed by the Bonferroni’s post-test was used to analyze the time-course plots. Differences with *p* < 0.05 were considered statistically significant.

## 3. Results

### 3.1. CTX Working Dose

Crotoxin induces several harmful effects, which vary according to the target organ studied, the experimental design, animal species, and toxin dose and route of administration [16,17,18,19,20]. To determine a CTX dose capable of promoting tissue damage, in the present study we performed dose-response experiments where mice were treated with CTX at 10–300 µg/Kg (s.c.) for 6 h.

**Figure 1:**
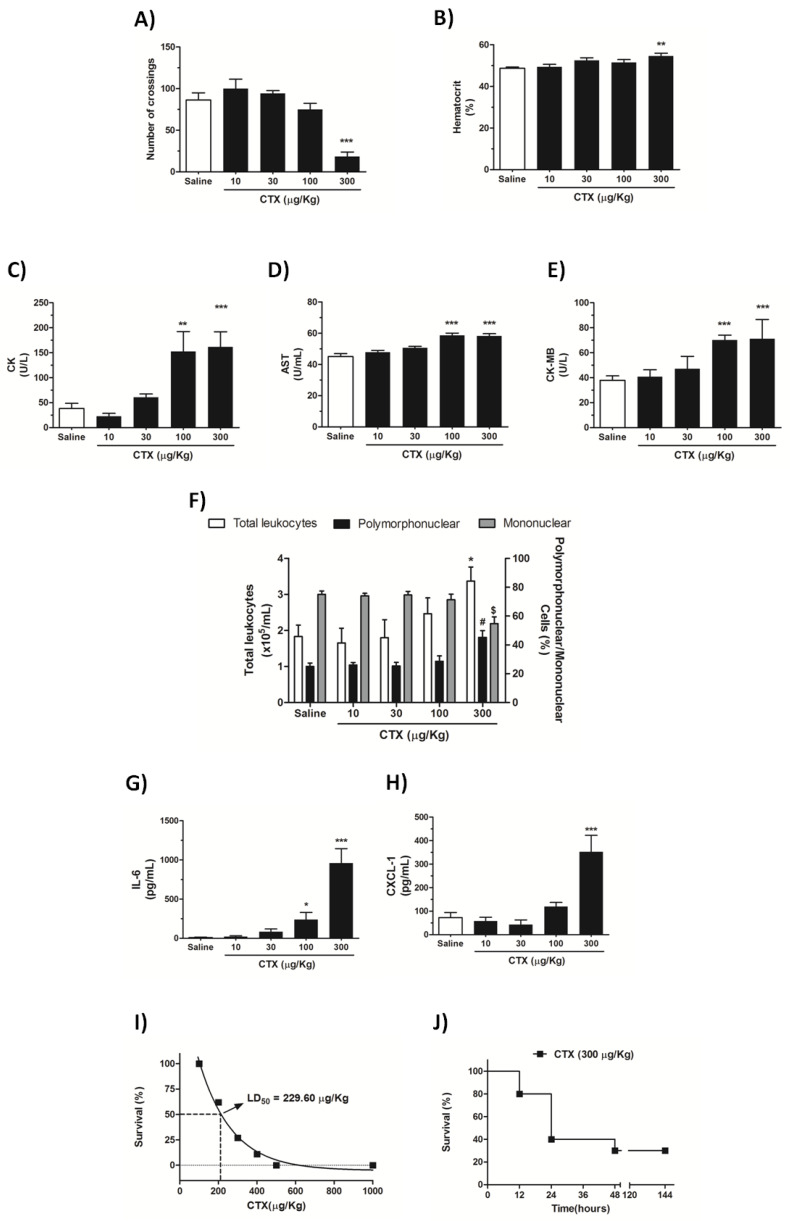
Dose-response toxicological effects of CTX. Mice were treated with different CTX doses (10–300 µg/Kg s.c.) and the biological parameters were evaluated at 6 h. (**A**) Open field test. (**B**) Hematocrit. (**C**) Serum creatine kinase (CK). (**D**) Serum creatine kinase-MB (CK-MB). (**E**) Serum aspartate aminotransferase (AST). (**F**) Total and differential leukocyte counting in dorsal air pouch fluid. (**G**) IL-6 levels in dorsal air pouch fluid. (**H**) CXCL-1 levels in dorsal air pouch fluid. (**I**) Determination of LD_50_. (**J**) Time-course survival profile. The results are representative from two-independent experiments (*n* = 6–7). ^#^ *p* < 0.05, ^$^ *p* < 0.05, * *p* < 0.05, ** *p* < 0.01, *** *p* < 0.001 compared with animals treated with saline (control)—One-way ANOVA followed by Tukey’s multiple comparison test multiple comparison test.

Compared with control mice, animals treated with 300 µg/Kg CTX exhibited reduced exploratory activity, as evidenced by the open field test analysis of locomotor alterations (Figure 1A), and a slightly increased hematocrit level, as evidenced by hematological analysis (Figure 1B). The serum levels of CK and CK-MB (biomarkers of muscle and heart tissue damage, respectively) and AST (a marker of liver damage) were increased in mice treated with 100 and 300 µg/Kg CTX (Figure 1C–E). Analysis of local inflammation in the dorsal air pouch cavity fluid evidenced increased infiltration of polymorphonuclear cells and production of the inflammatory mediators IL-6 and CXCL-1 in mice treated with the highest CTX dose (300 µg/Kg) (Figure 1F–H). In general, the CTX dose of 300 µg/Kg induced alterations in all the biological parameters evaluated; this dose is 1.3-fold greater than the determined LD_50_ of 229.6 µg/Kg (Figure 1i). The survival profile revealed that the toxin was lethal to approximately 20%, 60%, and 70% of the animals at 12, 24, 48 h, respectively (Figure 1I). Based on the set of results obtained so far, the CTX dose of 300 µg/Kg s.c. and the time treatment of 12 h—when the survival rate was ~80% (Figure 1J)—were selected for further investigations on how the toxin affects the respiratory system.

### 3.2. CTX Induces Lung Alterations

**Figure 2:**
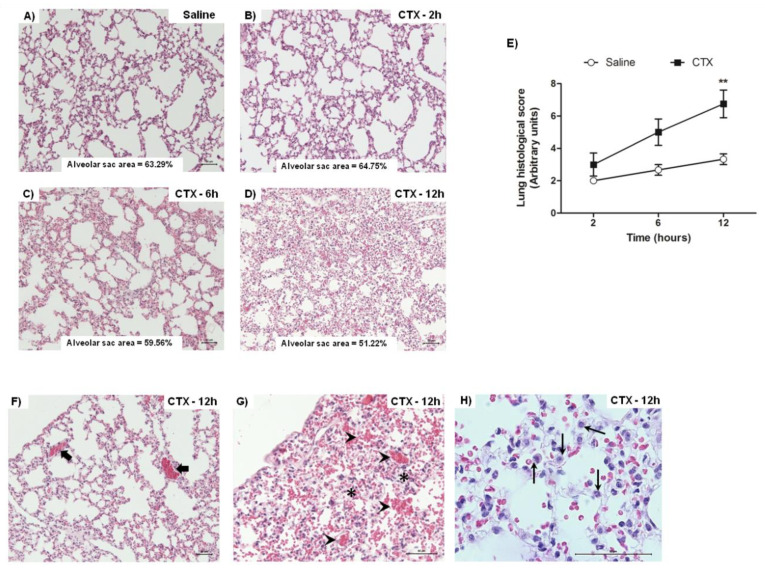
Histological alterations in mice lung induced by CTX. Representative sections of lungs from mice treated with saline or CTX (300 µg/Kg s.c.) for 2, 6, or 12 h. (**A–D**) Images from HE-stained sections were captured at 20X magnification (scale is represented in the image) and alveolar sac area was calculated using the IM-50 software. (**E**) Lung injury score was graded from 0–3 for each animal, and the result is representative of the mean of injury score. (**F**–**H**) Lung alterations after 12 h of CTX treatment: vascular congestion (thick arrow), edema (asterisk), alveolar hemorrhage (arrow head), and leukocyte infiltrate (thin arrow). ** *p* < 0.01 vs. saline-treated mice (control)—two-way ANOVA followed by the Bonferroni’s post-test (*n* = 3 animals/group).

Morphological analysis of lungs from animals treated with CTX for 2, 6, and 12 h revealed reduction of alveolar sac area and increased septum wall thickness, as compared with saline-treated animals (control) (Figure 2A–D). The lung histological score increased with time and was significantly different from the control at 12 h after CTX injection, indicating that lung damage was time-dependent (Figure 2E). At this treatment time, the presence of edema, vascular congestion, and –alveolar hemorrhage (Figure 2F–H), as well as leukocyte infiltration (especially of polymorphonuclear leukocytes) (Figure 2H), foamy macrophages, and hyperemia (data not shown) was also more evident.

**Figure 3:**
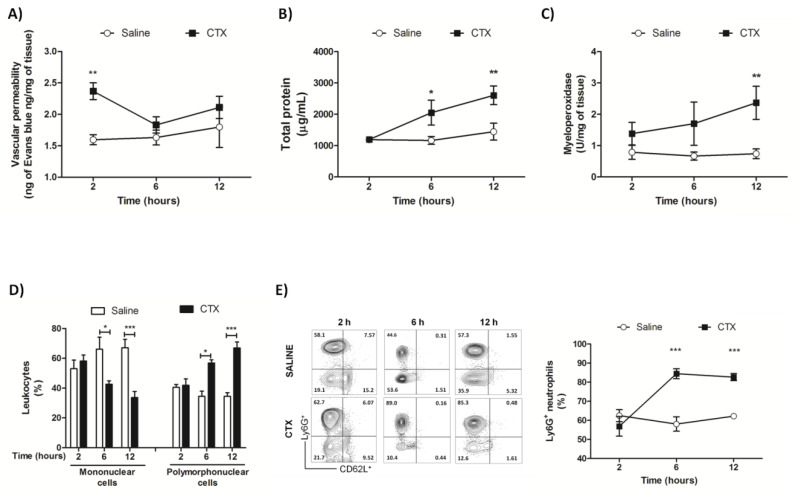
Lung alterations detected in lung homogenates from CTX-treated mice. The animals were treated with CTX (300 µg/Kg s.c.) for 2, 6, and 12 h, euthanized, and their lungs were removed and homogenized for the assays. (**A**) Vascular permeability was evaluated by injecting Evans blue solution 30 min before euthanasia. (**B**) Total protein concentration. (**C**) Myeloperoxidase activity. (**D**) Differential leukocyte counting from cytospin smears. (**E**) Flow cytometry data are summarized in the representative contour plots and line plots showing kinetics of increase of Ly6G^+^ cells. The results from (**A**–**C**) are representative from two independent experiments (*n* = 6–7 animals/group), while the results from (**D**–**E**) are representative from one independent experiment (*n* = 5 animals/group). * *p* < 0.05, ** *p* < 0.01, and *** *p* < 0.001 vs. saline-treated animals (control) from the respective time group—two-way ANOVA followed by the Bonferroni’s post-test.

The findings from morphological analysis guided determination of vascular permeability, total protein concentration, myeloperoxidase activity, and leukocyte infiltration in lung homogenates. Lung vascular permeability increased only at 2 h after CTX administration (Figure 3A). At 6 and 12 h of treatment with CTX, total protein concentration (Figure 3B) increased as a function of time and were significantly different from those detected in the control group. The time course myeloperoxidase activity increasing (Figure 3C) was followed by augmentation of leukocyte infiltration of polymorphonuclear cells (Figure 3D) and reflected by increased percentage of single Ly6G^+^ neutrophil population (Figure 3E), corroborating data from the increased number of granulocytes. Treatment with CTX did not alter the percentage of double Ly6G^+^ CD62L^+^ cells (data not shown).

Next, we analyzed the kinetics of leukocyte infiltration profile in bronchoalveolar fluid (BALF) and dorsal skin pouch fluid (PF). No neutrophil infiltration was detected in BALF from CTX-treated mice, at all experimental periods (data not shown), indicating that leukocyte infiltration was restricted to lung parenchyma. Leukocyte infiltration into PF increased at 6 h after CTX administration, but returned to basal levels at 12 h of treatment; this infiltrate was mainly composed of Ly6G^+^ polymorphonuclear cells (Supplementary Figure S1A–C).

### 3.3. CTX Elevates the Levels of Inflammatory Mediators and Impairs Pulmonary Function

**Figure 4:**
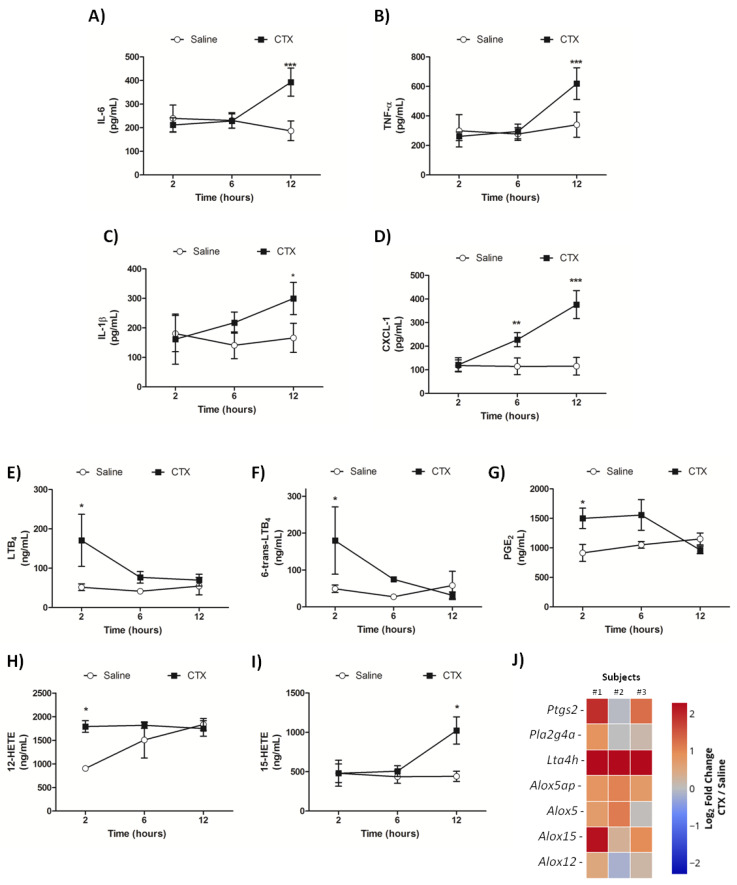
CTX-induced production of inflammatory mediators in mice lung. The animals were treated with CTX (300 µg/Kg s.c.) for 2, 6, and 12 h, euthanized, and their lungs were removed and homogenized for the quantification of pro-inflammatory cytokines/chemokines (**A**–**D**) and eicosanoids (**E**–**I**) using ELISA and LC-MS/MS, respectively. (**A**) Interleukin 6 (IL-6). (**B**) Tumor necrosis factor α (TNF-α). (**C**) Interleukin 1β (IL-1β). (**D**) Keratinocyte-derived chemokine (CXCL-1). (**E**) Leukotriene B_4_ (LTB_4_). (**F**) 6-*trans*-Leukotriene B_4_ (6-*trans*-LTB_4_). (**G**) Prostaglandin E_2_ (PGE_2_). (**H**) 15-Hydroxyeicosatetraenoic acid (15-HETE). (**I**) 12-Hydroxyeicosatetraenoic acid (12-HETE). (**J**) Gene expression profile in mice lung after 2 h of treatment with CTX, assessed by qRT–PCR. Data were expressed as log2 fold-change compared with the control (saline) (*n* = 3). The results from (**A**–**D**) are representative from two independent experiments (*n* = 6–7), while the results from (**E**–**I**) are representative from one independent experiment (*n* = 4 animals/group). * *p* < 0.05, *** *p* < 0.001 vs. saline treated animals (control) from the respective time group—two-way ANOVA followed by the Bonferroni’s post-test.

We analyzed the kinetics of cytokine, chemokine, and lipid mediator release in mice lung homogenates. The levels of the pro-inflammatory cytokines IL-1β, IL-6, and TNF-α increased at 12 h of treatment with CTX (Figure 4A–C). The levels of CXCL-1, which is a crucial mediator of neutrophil recruitment, increased at 6 and 12 h after CTX injection (Figure 4D).

Quantification of cytokines/chemokines in mice BALF and PF revealed that CTX injection did not alter the levels of IL-1β, IL-6, TNF-α, and CXCL-1 in BALF during the studied period of 12 h (data not shown). Considering that lung homogenates are composed of both lung tissue and BALF, and that the inflammatory markers remained unaltered in BALF, our findings indicate that the aforementioned lung alterations are exclusive to lung parenchyma, i.e., they do not occur in bronchoalveolar cavity. IL-6 levels in PF raised in a time-dependent manner up to 6 h after CTX administration, but returned to basal levels at 12 h of treatment. In contrast, the highest CXCL-1 levels in PF were detected at 2 h of CTX injection, and gradually declined up to basal levels at 12 h of treatment. The PF levels of TNF-α and IL-1β were not altered by CTX injection (Supplementary Figure S1D–G). Together, these findings illustrate a contrasting profile of cytokine/chemokine production in lungs and local dorsal skin in CTX-treated animals.

As the enzymatic phospholipase A_2_ activity of CTX mediates the production of signaling molecules, including eicosanoids that are associated with several biological effects induced by the toxin [21,22,23], in this study we examined the kinetics of lipid mediator production in mice lungs using a LC-MS/MS approach. Compared with the control group, concentration of the eicosanoids LTB_4_, 6-*trans*-LTB_4_, PGE_2_, and 12-HETE increased at 2 h of CTX injection (Figure 4E–H), while concentration of 15-HETE increased at 12 h of CTX injection (Figure 4I) but concentration of the mediators 6-keto-PGE_2_, PGD_2_, PGB_2_, PGF_2_a, 6-keto-PGF_1_a, TXB_2_, 11-HETE, and 5-oxo-ETE remained unaltered after CTX injection (data not shown). Gene transcripts for eicosanoid metabolism enzymes were analyzed in random lung samples from mice treated with CTX for 2 h. The heatmap (Figure 4J) illustrates a tendency of increase in expression of genes *Ptgs-2* (COX-2), *Lta4h*, *Alox15*, *Alox5*, and protein FLAP (*Alox5ap* gene), which play crucial roles during production of the abovementioned eicosanoids.

**Figure 5:**
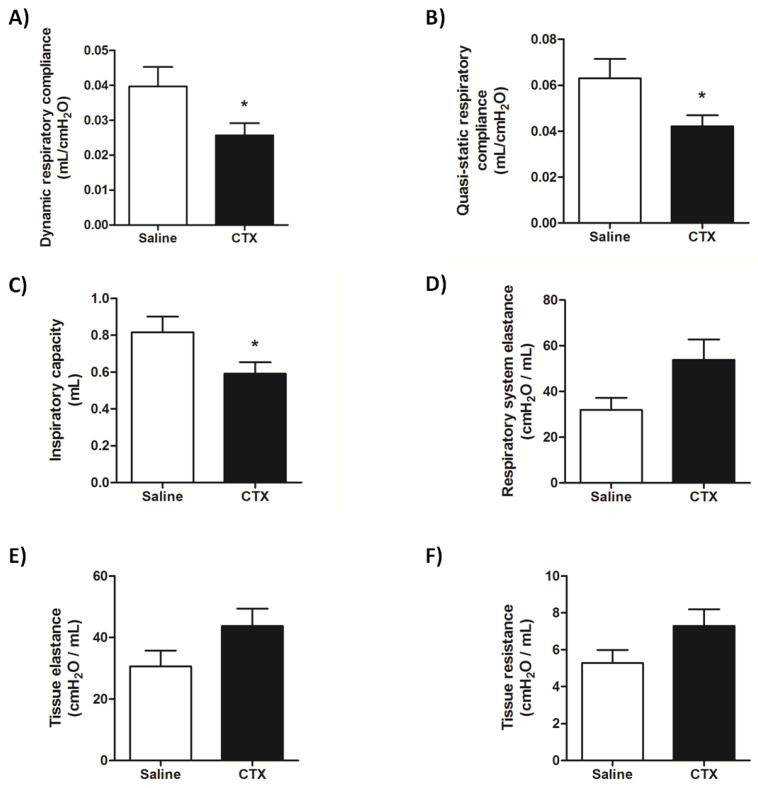
Lung mechanics in mice treated with CTX for 12 h. The respiratory mechanics parameters (**A**) Dynamic respiratory compliance, (**B**) Quasi-static respiratory compliance, (**C**) Inspiratory capacity, (**D**) Respiratory system elastance, (**E**) Tissue elastance, and (**F**) Tissue resistance were measured in vivo using a small animal ventilator, 12 h after CTX (300 µg/Kg s.c.) or saline (control) administration. The results are representative from two independent experiments (*n* = 8–10 animals/group). * *p* < 0.05 vs. control group—unpaired Student’s *t*-test.

Considering that CTX induced pathological alterations in lung, next we examined how the toxin affected lung mechanics in mice 12 h after its injection. Compared with the control group (saline), the respiratory system of CTX-treated mice exhibited reduced dynamic and quasi-static compliances (Figure 5A–B), indicating lung stiffness, as well as decreased inspiratory capacity (Figure 5C). In addition, the respiratory system and tissue elastance, and tissue resistance tended to increase in CTX-treated mice, but these parameters did not significantly differ from the control (*p* = 0.061, 0.110, and 0.108 respectively) (Figure 5D–F).

### 3.4. Indomethacin and Hexamethonium Mitigate CTX-Induced Lethality

**Figure 6:**
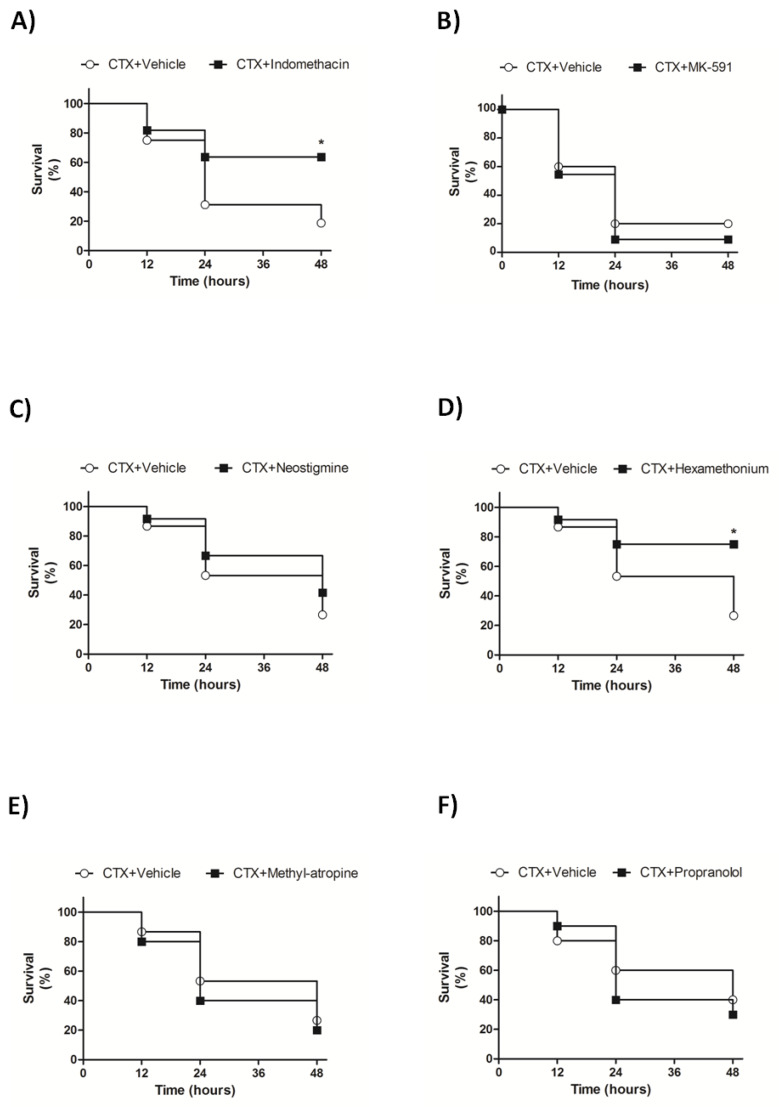
Role of eicosanoids metabolism and peripheral nervous system on survival of CTX-treated mice. Animals were treated with (**A**) Indomethacin (3 mg/Kg i.p.), (**B**) MK-591 (40 mg/Kg i.p.), (**C**) Neostigmine (0.1 mg/Kg i.p.), (**D**) Hexamethonium (10 mg/Kg i.v.), (**E**) Methyl-atropine (30 mg/Kg i.p.), and (**F**) Propranolol (5 mg/Kg i.p.) or their respective vehicles before CTX administration (300 µg/Kg). Survival rate was determined every 12 h for 48 h. Results are expressed as percentage of survival and are representative from two independent experiments (*n* = 13–16 animals/group). * *p* < 0.05—Mantel–Cox log-rank test.

Here we used drugs that act as antagonists or inhibitors of lipid mediator metabolism or peripheral nervous system pathways as the pharmacological approach to investigate the mechanisms by which CTX induced lethality. The COX-1 and -2 inhibitor indomethacin, but not the 5-lipoxygenase pathway inhibitor MK-591, increased the survival rate of CTX-treated animals (Figure 6A,B). Analysis of the peripheral neural effects involving nicotine pharmacology evidenced that the blocker of ganglionic nicotinic receptors hexamethonium, but not the acetylcholinesterase inhibitor neostigmine, increased the survival rate of CTX-treated animals (Figure 6C,D). In addition, neither the peripheral muscarinic antagonist methyl-atropine nor the adrenergic antagonist propranolol altered the survival rate of CTX-treated mice (Figure 6E,F). Therefore, these results clearly indicate that both COX-derived prostanoids and peripheral nicotinic receptors are associated with CTX-induced lethality.

### 3.5. Role of Hexamethonium And Indomethacin on CTX-Induced Lung Alterations

Our previous results revealed that CTX induced several morphological and functional alterations in lung parenchyma associated with inflammatory and vascular responses. Analysis of kinetics of CTX action evidenced that some alterations were clearly detected at the early (at 2 h) or late (at 12 h) phases of response to toxin administration. Considering that COX-derived prostanoids and nicotinic acetylcholine receptors were involved in CTX pathogenesis, here we examined how their antagonists indomethacin and hexamethonium, respectively, interfered with early and late phase lung alterations.

#### 3.5.1. Early Phase

**Figure 7:**
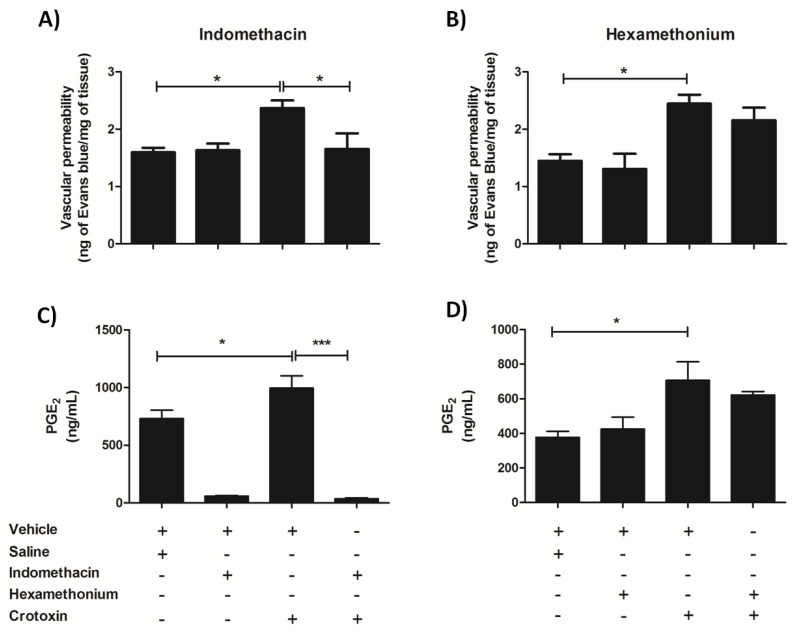
Effect of indomethacin and hexamethonium on CTX-induced early phase lung alterations. Mice were treated with indomethacin (3 mg/Kg i.p.; panels (**A**,**C**)), hexamethonium (10 mg/Kg i.v.; panels (**B**,**D**)), or their respective vehicles, before CTX (300 µg/Kg s.c.) administration. After 2 h, lungs were collected for (**A**,**B**) analysis of vascular permeability using the Evans blue extravasation assay and (**C**,**D**) quantification of prostaglandin E_2_ (PGE_2_) by ELISA. The results are representative from one independent experiment (*n* = 4 animals/group). * *p* < 0.05 and *** *p* < 0.001—one-way ANOVA followed by the Tukey’s multiple comparison test.

The early phase lung alterations, which were analyzed 2 h after CTX treatment, were marked by increased vascular permeability and release of eicosanoids (previous Figure 3A and Figure 4E–I, respectively). Indomethacin, but not hexamethonium, mitigated the CTX-induced enhancement of vascular permeability (Figure 7A,B)—as assessed by the Evans blue extravasation assay—and strongly lowered basal (saline/vehicle) and CTX-induced increase of lung PGE_2_ levels (Figure 7C,D). Altogether, these findings indicated that PGE_2_ participated in the early phase lung alterations induced by CTX.

#### 3.5.2. Late Phase

**Figure 8:**
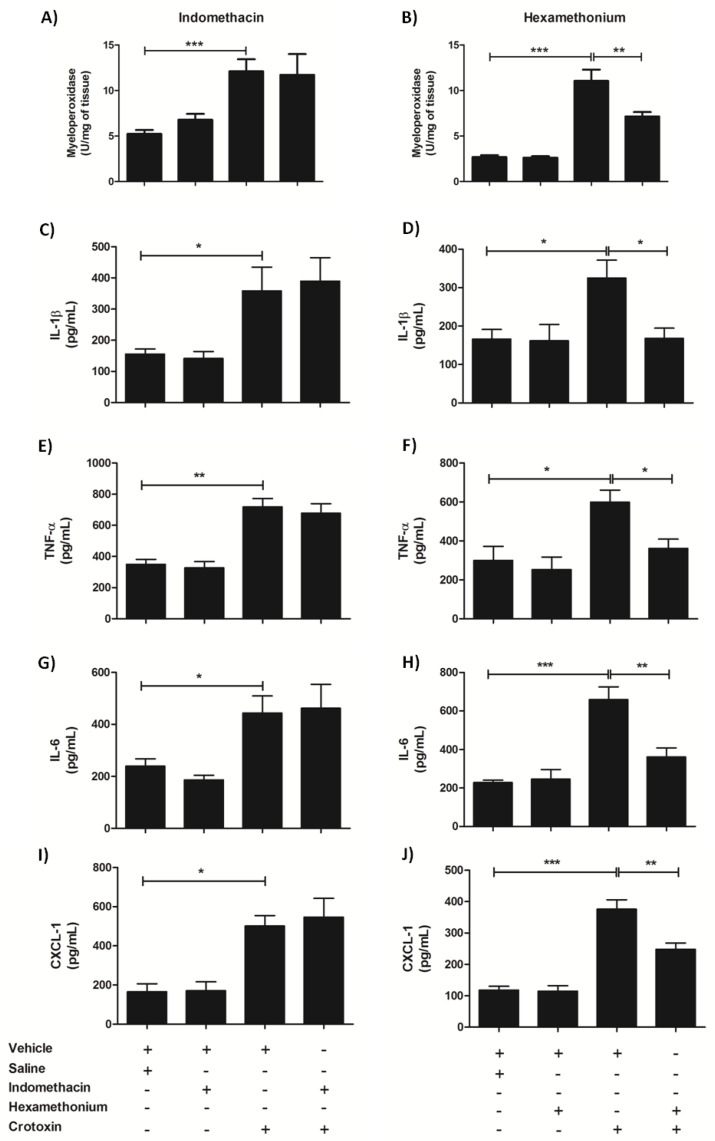
Effect of indomethacin and hexamethonium on ctx-induced late phase lung alterations. Animals were treated with indomethacin (3 mg/Kg i.p.) or hexamethonium (10 mg/Kg i.v.), or their respective vehicles, before CTX (300 µg/Kg s.c.) administration. After 12 h, lungs were collected for quantification of (**A**,**B**) Myeloperoxidase activity, (**C**,**D**) Interleukin 1β (IL-1β), (**E**,**F**) Tumor necrosis factor α (TNF-α), (**G**,**H**) Interleukin 6 (IL-6), and (**I**,**J**) Keratinocyte-derived chemokine (CXCL-1). The results are representative from two independent experiments (*n* = 6–7 animals/group). * *p* < 0.05, ** *p* < 0.01, and *** *p* < 0.001—one-way ANOVA followed by the Tukey’s multiple comparison test.

The late phase lung alterations, analyzed 12 h after CTX treatment, were characterized by increased myeloperoxidase activity and increased levels of pro-inflammatory cytokines/chemokines (previous Figure 3C and Figure 4A–D, respectively). The effects of indomethacin and hexamethonium were the opposite of those detected on the early phase lung alterations: the latter but not the former drug mitigated the CTX-induced enhancement of myeloperoxidase activity (Figure 8A,B) and levels of the inflammatory markers IL-1β (Figure 8C,D), TNF-α (Figure 8E,F), IL-6 (Figure 8G,H), and CXCL-1 (Figure 8I,J). These results pointed out the participation of peripheric nicotinic receptors in the late phase lung alterations.

We also investigated whether indomethacin and hexamethonium altered the levels of leukocyte infiltration and inflammatory mediators in PF. Compared with CTX-treated mice, animals treated with indomethacin prior to CTX injection exhibited increased levels of CXCL-1 and IL-6, and an almost significantly increased leukocyte infiltration level (Supplementary Figure S2A,C,E). Administration of hexamethonium before CTX injection did not alter the levels of the inflammatory parameters analyzed (Supplementary Figure S2B,D,F).

To continue analyzing the late phase inflammatory parameters, we determined the serum CK-MB levels at 12 h after CTX administration in mice pretreated with hexamethonium and indomethacin. Both drugs mitigated the CTX-induced increase in CK-MB levels (Supplementary Figure S3), suggesting that both COX metabolism and peripheral nicotinic receptors are associated with cardiovascular alterations induced by CTX.

### 3.6. Hexamethonium Mitigates CTX-Induced Lung Mechanics Impairment

**Figure 9:**
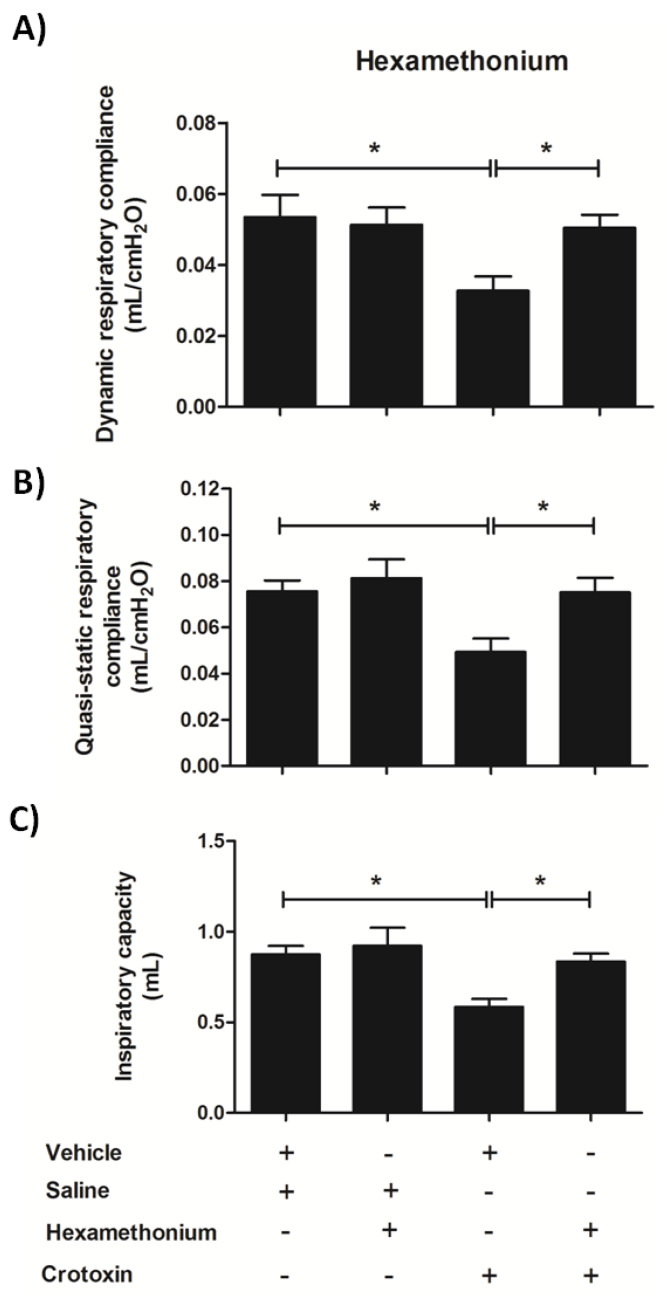
Effect of hexamethonium on lung mechanics of CTX-treated mice. Mice were treated with hexamethonium (10 mg/Kg i.v.) 30 min before CTX (300 µg/Kg s.c.) administration. After 12 h, we used a small animal ventilator to determine the respiratory mechanics parameters in vivo. (**A**) Respiratory system compliance. (**B**) Quasi-static compliance. (**C**) Inspiratory capacity. The results are representative from two independent experiments (*n* = 7–8 animals/group). * *p* < 0.05—one-way ANOVA followed Tukey’s multiple comparison test.

Based on the previous results that clearly demonstrated that hexamethonium effectively dampened CTX-induced late phase lung alterations, here we examined how the nicotinic blocker modulated the toxin-induced impairment of pulmonary function after 12 h of treatment. Pre-treatment with hexamethonium prevented the CTX-induced alterations in pulmonary mechanics, i.e., it improved quasi-static and respiratory system compliances (Figure 9A,B) and inspiratory capacity (Figure 9C) when compared with mice treated with CTX alone.

## 4. Discussion

The systemic effects of *C. durissus* venom are stronger than its local effects, and are mainly characterized by neurotoxicity, systemic myotoxicity, and respiratory and acute renal failure [24,25,26]. Respiratory failure only occurs in severe cases of envenomation by rattlesnake bite [27,28,29,30,31]. As the major venom component, CTX plays a significant role on *Crotalus* accidents, including neuromuscular blockade and systemic myotoxicity as the main toxicological effects, associated with other alterations.

To investigate how CTX affects respiratory physiology, we selected a dose that causes significant intoxication by evaluating some biological alterations, based on previous findings. CTX-induced locomotor alterations are associated with neuromuscular disturbances caused by neuromuscular blockade [16,17,32,33] and myotoxicity [20,34]. We found that subcutaneous injection of CTX at a dose of 300 µg/Kg significantly decreased mice exploratory activity at the open field test and increased serum CK, which indicate locomotor impairment and systemic myotoxicity. This toxin dose also effectively induces other previously reported biological alterations such as increased blood hematocrit [35], dorsal skin local inflammation [19], and increased levels of AST and CK-MB associated with liver and heart damage, respectively [18,36,37,38]. The LD_50_ found in the present work (229.6 µg/Kg s.c.) was relatively close to that reported by Brazil and colleagues [39] (177.5 µg/Kg, s.c.) in mice. The selected dose for the following experiments (300 µg/Kg) represented 1.3 LD_50_, and enabled a reliable characterization of the toxicological effects of CTX on the respiratory system.

The respiratory system is a complex arrangement of organs that promote the respiration, coordinated by neural control of respiratory muscles of pump (diaphragm, intercostals and abdominal) and bronchomotor tone (airway smooth muscles), and which the respiratory tract (composed of specialized tissues and cells) responsible for the maintenance of the architecture and gas exchange [40]. Lung disorders are characterized by alterations that impair airway, vessel or lung function, and are accompanied by changes in tissue morphology [41]. In the present study, histological analysis of lung from CTX-treated mice evidenced progressive tissue damage characterized by the presence of edema, vascular congestion, alveolar hemorrhage, and leukocyte infiltration, with the highest histological scores at 12 h after CTX injection. These results agree with previous findings on lung morphological changes after C.d.t. and *C. durissus cascavella* whole venom administration, especially the presence of perivascular edema, diffuse hemorrhage, and leukocyte infiltration up to 24 h after venom administration [42,43].

We used lung homogenates to investigate deep tissue alterations, including vascular permeability, total protein concentration, myeloperoxidase activity, leukocyte infiltration profile, and levels of pro-inflammatory cytokines and lipid mediators. We also analyzed the leukocyte infiltration profile and concentration of pro-inflammatory cytokines in BALF, but we did not detect alterations in these inflammatory markers (data not shown), indicating that CTX selectively acted on lung parenchyma rather than bronchoalveolar cavity.

An overall analysis of the time-course of lung injuries evidenced two patterns: early phase alterations within the first two hours, and late phase alterations that begun (or not) at 6 h and peaked after 12 h. The early phase was marked by a transient increase in lung vascular permeability, identified by tissue accumulation of Evans blue. Acute lung injuries are characterized by an early phase increase in vascular permeability, whose evolution can impair respiratory function [44,45]. CTX also upregulated the cyclo- and lipoxygenase pathways of eicosanoids production. The levels of the enzymatic product PGE_2_ were augmented. Expression of the 15-lipoxygenase (*Alox15*), but not the 12-lipoxygenase (*Alox12*) gene was upregulated and associated with increased 12- and 15-HETE levels. Elevation of LTB_4_ levels was accompanied by a rise in gene expression of the 5-lipoxygenase complex enzyme (*ALOX5*), its associated protein 5-lipoxygenase activated protein (FLAP—*ALOX5ap*)—which is required for 5-lipoxygenase activity [46], and LTA_4_ hydrolase (*Lta4h*); both enzymes, 5-lipoxygenase and LTA_4_ hydrolase, catalyze LTB_4_ biosynthesis [47].

We detected increased levels of 6-*trans*-LTB_4_—a non-enzymatic product from LTA_4_—along with gene expression of cytosolic PLA_2_ (*Pla2g4a*)—an important enzyme responsible for arachidonic acid release [48]. The aforementioned eicosanoids are common mediators of inflammatory response that can participate in lung impairment [49,50,51,52], but this hypothesis will be discussed further. Another plausible assumption for the participation of eicosanoids in CTX-induced lung alteration relies on the PGE_2_-mediated increase of vascular permeability [53]. Additionally, PGE_2_ is a known bronchoconstrictor agent with a direct effect on airways smooth muscle [54], and possibly being responsible for minor effect on airway impairment.

The involvement of eicosanoids in the biological effects of CTX has been widely investigated. For instance, the literature reports that PLA_2_ enzymatic activity of CTX towards cell membrane phospholipids releases free arachidonic acid, and mediates the toxin-induced biosynthesis of lipoxin A_4_ via the lipoxygenase pathway, and of the prostanoids PGE_2_, PGD_2_, and 15-d-PGJ_2_ via participation of COX-1 and Ca^2+^-independent PLA_2_. These mediators are associated with several biological effects of the toxin, such as modulation of leukocyte function and anti-inflammatory and immunosuppressive responses [21,55,56,57].

The late phase lung injuries were characterized by inflammatory alterations that peaked 12 h after CTX administration, including neutrophil infiltrate composed of Ly6G^+^ cells, associated with increased levels of myeloperoxidase activity, an indirect indicator of tissue neutrophil content [58], total proteins, and the pro-inflammatory cytokines/chemokines IL-6, TNF-α, IL-1β, and CXCL-1. High CTX doses promote local and systemic pro-inflammatory effects, such as paw edema, local and systemic muscle necrosis with neutrophil infiltration, and blood neutrophilia associated with increased serum levels of IL-6 and IL-10 [31,59,60,61]. Although CTX induced an inflammatory pattern with increased leukocyte infiltration and production of pro-inflammatory cytokines and lipid mediators, its intensity was not as strong as that found in infectious disease [62] and was restricted to parenchyma; these findings unveil a new pathophysiologic scenario in the experimental model studied herein.

Lung mechanics is an important feature in respiratory physiology that is associated with elastic and resistive properties of lung tissue [63]. Hence, alterations in parenchymal tissue can induce biomechanical loss of function and result in respiratory impairment [64]. Considering the CTX-induced injury and morphological alterations in mice lungs, we used the forced oscillation technique [65] to assess invasive lung function. We found that CTX-treated mice exhibited diminished dynamic and quasi-static respiratory compliances—two parameters that are associated with the lung ability to expand during inspiration and active expiration, and whose decrease is associated with lung stiffness [66]. Furthermore, tissue changes that lead to lung stiffness and make the respiratory system to work harder culminate in reduced inspiratory capacity [67,68]. We hypothesize that the lung tissue morphological injuries caused by CTX accounted for the lung stiffness and reduced inspiratory capacity in our study.

Next, we investigated the mechanisms by which CTX caused pulmonary impairment. CTX is a β-neurotoxin that induces neuromuscular blockade by inhibiting presynaptic acetylcholine release and postsynaptic desensitization of nicotinic receptors in neuromuscular junction, resulting in a flaccid paralysis [16,69,70,71]. Additionally, the PLA_2_ enzymatic activity of CTX is associated with (i) several toxicological effects, such as myotoxicity characterized by degradation of cell membrane phospholipids and muscle tissue necrosis; and (ii) production of lipid mediators like PGE_2_, which are associated with myotoxicity, neurotoxicity, and activation of immune responses [21,55,72,73,74,75]. In this sense, we examined the participation of peripheral nervous system and lipid mediators in CTX-induced pulmonary alterations and lethality. Pretreatment of mice with indomethacin and hexamethonium prior to CTX injection reduced the toxin lethality rate. Indomethacin is a COX-1 and -2 inhibitor that suppresses the biosynthesis of prostaglandins [76], while hexamethonium is a nicotinic acetylcholine receptor antagonist acting promiscuously on both ganglionic and less effectively on neuromuscular junction from peripheral nervous system, [77,78]. We also observed that methyl-atropine (muscarinic acetylcholine receptor antagonist) and propranolol (β-adrenergic receptor antagonist) did not change the CTX-induced lethality rate, as well as neostigmine (acetylcholinesterase inhibitor), probably due to the toxin capacity to deplete acetylcholine vesicles in nerve cholinergic endings [79]. These results suggest that the toxicological effects could be associated with the modulation of cholinergic transmission involving nicotinic receptors, but not muscarinic. Another harmful effect of CTX related to participation of both prostanoids and peripheral nervous system is associated with the toxin capacity to induce cardiovascular alterations. The toxin causes systemic hypotension in dogs and rabbits when the vagus nerve response is stimulated, since it is a parasympathetic-related controller of heart and lung function [35,80]. An in vitro study using the Langendorff model has demonstrated that CTX weakens heart contractile force and increases CK release [72]. Indomethacin reverts both effects, indicating the participation of COX-derived mediators, such as PGE_2_, in these events [72]. In the present work, CTX increased serum CK-MB levels, and pretreatment with hexamethonium and indomethacin mitigated such rise. As CK-MB is a biomarker of heart damage, our findings strongly indicate that CTX-induced cardiovascular alterations are associated with production of COX-derived prostanoids and modulation of nicotinic receptors, and can account for the toxin-induced respiratory compromise.

Analysis of the interference of hexamethonium and indomethacin on both early and late phase lung alterations revealed that they have divergent modulatory actions. Indomethacin, but not hexamethonium, lowered the CTX-induced lung vascular permeability and PGE_2_ production. Vascular permeability occurs in the early phase of lung diseases and is associated with lung tissue alterations such as edema [44,45,81]. PGE_2_ also elicits vasodilation and thereby increases vascular permeability by acting on EP2 and EP4 receptors [53]. Considering previous studies on CTX biodistribution that report that the toxin reaches lung tissues after 10 min of i.v. administration [82,83], we can assume that, in the early phase, (i) CTX directly acted on lung tissues in order to induce the production of lipid mediators; and (i) PGE_2_ mediated the increased lung vascular permeability. Regarding the late phase lung alterations, characterized by an inflammatory response, hexamethonium but not indomethacin mitigated the CTX-induced increase in lung myeloperoxidase activity and in the levels of the pro-inflammatory cytokines/chemokines IL-1β, IL-6, TNF-α, and CXCL-1. Altogether, these findings indicate that CTX-induced PGE_2_ production was not involved in lung inflammation, and that hexamethonium acted as peripheral nervous system nicotinic blocker and did not have a direct anti-inflammatory action; hence, the CTX-induced lung inflammatory response seemed to be associated with a secondary effect of the toxin.

To examine whether CTX induces lung inflammation directly or indirectly, we compared data from local inflammatory profile using the dorsal air pouch model with data from lung tissue homogenates. Both local and tissue inflammatory responses were characterized by the presence of neutrophil infiltration and pro-inflammatory cytokines/chemokines, which peaked at 6 and 12 h in pouch fluid and lung tissue, respectively. Such kinetic difference can be explained by CTX biodistribution, but it does not exclude the possibility of a direct toxin action on the lungs. The increased levels of TNF-α and IL-1β in lung tissue but not in pouch fluid indicated that these compartments had different inflammation patterns. In addition, hexamethonium had no effect on total leukocyte counting and IL-6 and CXCL-1 levels in pouch fluid, but mitigated the CTX-induced increase in these parameters in mice lungs. Interestingly, pre-treatment with indomethacin worsened the CTX-induced local inflammatory response, suggesting that PGE_2_ favors resolution of the inflammatory process. Although it is known that PGE_2_ plays an anti-inflammatory role [50] and CTX also presents anti-inflammatory and immunosuppressive properties [84], the resolution effect of PGE_2_ associated with CTX has never been described. Therefore, lung inflammation in CTX-treated mice was not associated with a direct effect of the toxin on the lungs.

Treatment with hexamethonium also significantly mitigated the CTX-induced impairment of lung function, which was another late phase alteration; in particular, it restored the dynamic and quasi-static respiratory compliances and inspiratory capacity. The neurophysiology of respiration involves the participation of cholinergic transmission from i) parasympathetic nerves that activates mAChRs, present on airway smooth muscle and blood vessels, causing bronchoconstriction and vasodilatation, and ii) and motor nerve fibers responsible for promoting diaphragm and intercostals muscles activation mediated by nAChRs [85,86]. Consequently, a possible mechanism of CTX lung toxicity would involve the modulation of nicotinic receptors, at ganglionar and/or neuromuscular junction levels, of cholinergic fibers of respiratory muscles. Our data clearly support the hypothesis that CTX induced respiratory impairment by triggering peripheral paralysis of airway muscles, including the diaphragm, which can be associated with development of lung hypoxia. CTX-induced respiratory paralysis in rabbits promotes severe acidosis and hypoxia, as demonstrated by decreased blood pH and pO_2_, and increased pCO_2_ [80]. Some alterations that occur in several pathologies associated with neuromuscular paralysis and hypoxia are the development of an inflammatory response comprising neutrophil infiltration, release of pro-inflammatory mediators, lung tissue fibrosis, decreased lung compliance, and increased lung resistance [87,88,89]. Therefore, the CTX-induced late phase alterations were not associated with a direct toxin effect on the lungs, but with a neuromuscular blockade that triggered airway muscle paralysis and promoted a hypoxemic condition.

## 5. Conclusions

Here we report for the first time the CTX-induced lung alterations and their implications on the respiratory function, as well as the mechanisms involved. The toxin causes acute respiratory failure characterized by early and late phase lung alterations. The early phase is marked by a direct CTX action on lung tissue that increases the production of lipid mediators, among which PGE_2_ is supposed to mediate the increased vascular permeability. Additionally, the capacity to modulate lung cholinergic transmission via nicotinic receptors, possible at ganglionic and neuromuscular levels, induced a set of late phase alterations characterized by a moderate, but consistent, inflammatory response associated with morphological alterations characterized by tissue septum wall thickening, edema, hemorrhage, and decreased alveolar sac areas. Together, these effects impair lung mechanical function—more specifically, lung compliance and inspiratory capacity are decreased—and lead to animal death. Considering the CTX-induced mice lung impairment, we conclude that respiratory failure is the determinant factor for murine death. The findings reported herein highlight the impact of CTX on respiratory compromise, and introduce the use of nicotinic blockers and prostaglandin biosynthesis pathway inhibitors as possible symptomatic therapy to envenomed patients.

The present work deeply investigated the CTX-induced respiratory impairment, which is an important pathological condition of rattlesnake-envenomed patients. Additional studies on the toxicological effects of CTX are required due to its dual role: (1) as the major component, the toxin is a key player of pathological events induced by C.d.t. venom, whose understanding may help to develop therapeutic interventions to envenomed patients thru nicotinic acetylcholine antagonists drugs; (2) the toxin has promising medicinal applications due to its anti-inflammatory, immunosuppressive, antitumor, and analgesic properties [84,90,91,92]. The potential medicinal application of CTX, as demonstrated in phase I clinical trials against cancer [18,93], stresses the importance of analyzing the side effects of the toxin that may occur in possible cases of intoxication during its therapeutic use.

## Supplementary Materials

The following are available online at https://www.mdpi.com/2218-273X/10/5/794/s1, Figure S1: Inflammatory parameters in air pouch fluid from CTX-treated mice; Figure S2: Effects of indomethacin and hexamethonium on air pouch inflammation in CTX-treated mice; Figure S3: Modulation of serum CK-MB levels by hexamethonium and indomethacin in CTX-treated mice.
