# Supplementary material for: Crotoxin-Induced Mice Lung Impairment: Role of Nicotinic Acetylcholine Receptors and COX-Derived Prostanoids
Source: Biomolecules. 2020 May 20;10(5):794. doi: 10.3390/biom10050794 (PMC7277605; doi:10.3390/biom10050794)
Supplement: Supplementary file 1 [file biomolecules-10-00794-s001.pdf]

Appendix A

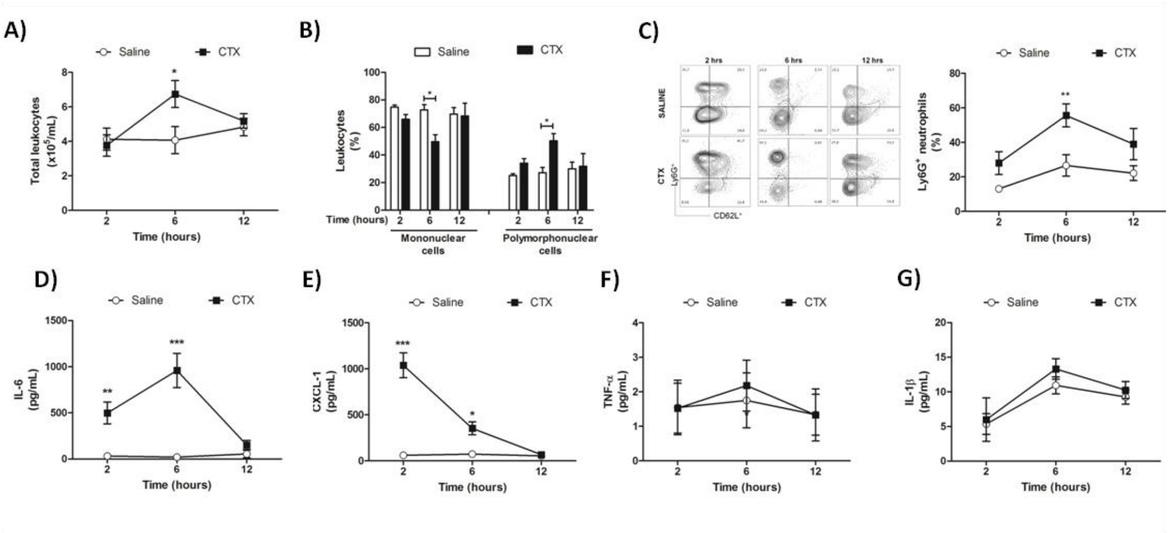

Supplementary Figure S1. Inflammatory parameters in air pouch fluid from CTX-treated mice. Pouch fluid (PF) were collected from mice after 2, 6, and 12 h of CTX s.c. injection to analyze the inflammatory parameters. (**A**) Total leukocyte counting. (**B**) Differential leukocyte counting. (**C**) Flow cytometry data are summarized in the representative contour plots and line plots showing kinetics of alteration in the $Ly6G^+$ cell population. (**D**) Interleukin 6 (IL-6). (**E**) Keratinocyte-derived chemokine (CXCL-1). (**F**) Tumor necrosis factor α (TNF-α). (**G**) Interleukin 1β (IL-1β). The results are representative from two independent experiments (n = 6–7). * $P < 0.05$, ** $P < 0.01$, *** $P < 0.001$ vs saline-treated animals (control) from the respective time group - two-way ANOVA followed by the Bonferroni's post-test.

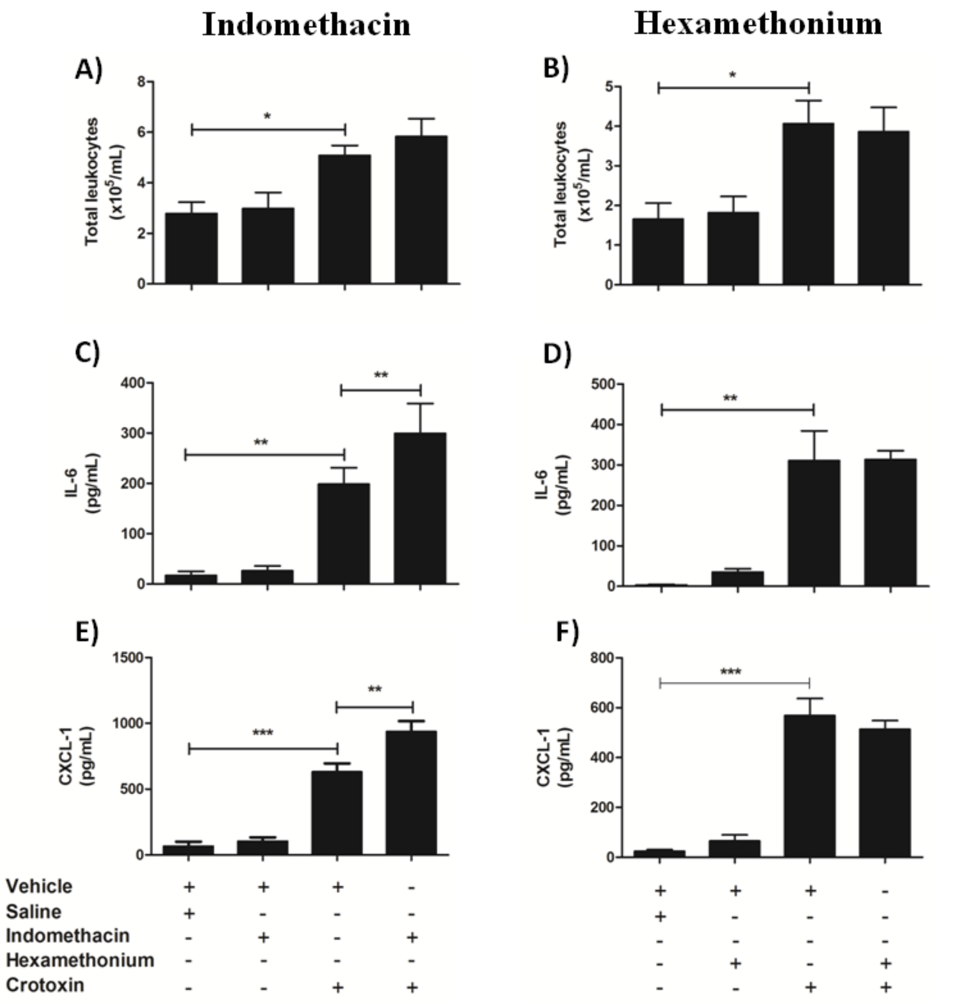

Supplementary Figure S2. Effects of indomethacin and hexamethonium on air pouch inflammation in CTX-treated mice. The animals were treated with hexamethonium (10 mg/Kg i.v., 30 min prior to CTX) or indomethacin (3 mg/Kg i.p., 4 h prior to and 30 min after CTX) before CTX (300 μg/Kg s.c.) administration. After 6 h, the following inflammatory parameters were analyzed in air pouch: (**A**-**B**) Total leukocyte counting, (**C**-**D**) Interleukin 6 (IL-6), and (**E**-**F**) Keratinocyte-derived chemokine (CXCL-1) levels. The results are representative from two independent experiments (*n* = 6 animals/group). * *P < 0.05*, ** *P < 0.01*, *** *P < 0.001* vs. control (vehicle/saline) group—One-way ANOVA followed by the Tukey's multiple comparison test.

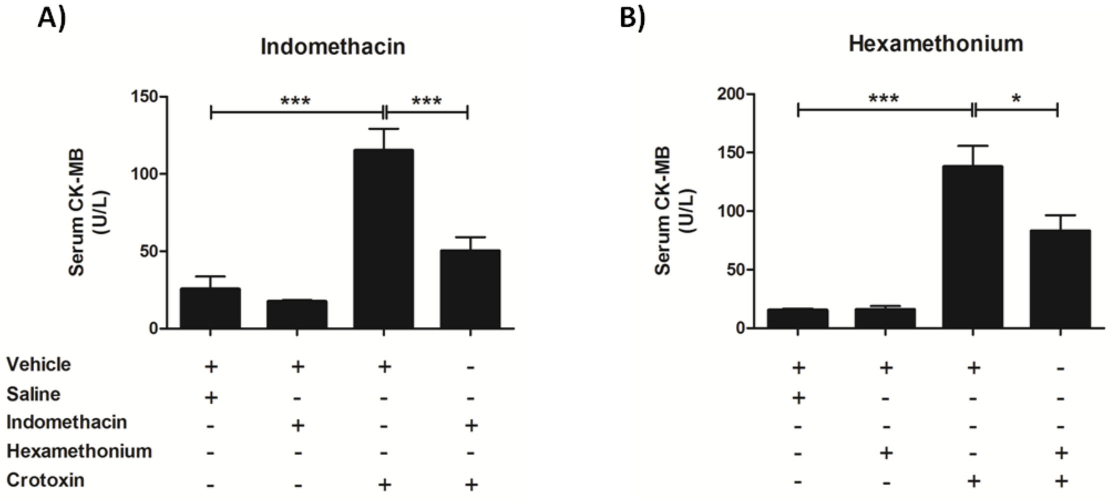

Supplementary Figure S3. Modulation of serum CK-MB levels by hexamethonium and indomethacin in CTX-treated mice. Animals were treated with (**A**) indomethacin (3 mg/Kg i.p.) or (**B**) hexamethonium (10 mg/Kg i.v.), or their respective vehicles, before CTX (300 μg/Kg s.c.) administration. After 12 h, blood was collected for serum CK-MB quantification. The results are representative from two independent experiments (*n* = 6–7 animals/group). * *P < 0.05*, ** *P < 0.01*, and *** *P < 0.001*—One-way ANOVA followed by the Tukey's multiple comparison test.
